# Offering self-administered oral HIV testing to truck drivers in Kenya to increase testing: a randomized controlled trial

**DOI:** 10.1080/09540121.2017.1360997

**Published:** 2017-08-21

**Authors:** Elizabeth A. Kelvin, Gavin George, Eva Mwai, Eston Nyaga, Joanne E. Mantell, Matthew L. Romo, Jacob O. Odhiambo, Lila Starbuck, Kaymarlin Govender

**Affiliations:** a Department of Epidemiology and Biostatistics & Institute for Implementation Science in Population Health, CUNY Graduate School of Public Health and Health Policy, City University of New York, New York, NY, USA; b Health Economics and HIV and AIDS Research Division, University of KwaZulu-Natal, Durban, South Africa; c North Star Alliance, Nairobi, Kenya; d HIV Center for Clinical and Behavioral Studies, Department of Psychiatry, Division of Gender, Sexuality and Health, New York State Psychiatric Institute & Columbia University, New York, NY, USA

**Keywords:** Diagnostic tests, HIV, HIV testing, Kenya

## Abstract

We conducted a randomized controlled trial among 305 truck drivers from two North Star Alliance roadside wellness clinics in Kenya to see if offering HIV testing choices would increase HIV testing uptake. Participants were randomized to be offered (1) a provider-administered rapid blood (finger-prick) HIV test (i.e., standard of care [SOC]) or (2) a Choice between SOC or a self-administered oral rapid HIV test with provider supervision in the clinic. Participants in the Choice arm who refused HIV testing in the clinic were offered a test kit for home use with phone-based posttest counseling. We compared HIV test uptake using the Mantel Haenszel odds ratio (OR) adjusting for clinic.

Those in the Choice arm had higher odds of HIV test uptake than those in the SOC arm (OR = 1.5), but the difference was not statistically significant (*p* = 0.189). When adding the option to take an HIV test kit for home use, the Choice arm had significantly greater odds of testing uptake (OR = 2.8, *p* = 0.002). Of those in the Choice arm who tested, 26.9% selected the SOC test, 64.6% chose supervised self-testing in the clinic, and 8.5% took a test kit for home use.

Participants varied in the HIV test they selected when given choices. Importantly, when participants who refused HIV testing in the clinic were offered a test kit for home use, an additional 8.5% tested. Offering truck drivers a variety of HIV testing choices may increase HIV testing uptake in this key population.

## Introduction

Truck drivers in sub-Saharan Africa are at high risk for HIV (Ojo et al., 2011). This may be, in part, because mobility forces couples to be apart, leading to the use of commercial sex services which cluster around transportation routes (International Labor Organization, 2005; Lafort et al., 2010; Regondi, George, & Pillay, 2013). A 1991 study among truck drivers in Kenya reported that 61% visited female sex workers (FSWs), only 32% had ever used condoms (Bwayo et al., 1991), and 18% tested HIV+ (Bwayo et al., 1991). Another study in Kenya 1993–1997 found that HIV incidence among truck drivers was significantly higher than among stationary staff at the same company (i.e., administrators, mechanics) (Rakwar et al., 1999), and a 2003–2004 survey of 1896 long-distance truck drivers in South Africa found 26% HIV prevalence, with a dose-response relationship with time on the road (Delany-Moretlwe et al., 2014). Other studies among truck drivers in Africa also found high HIV prevalence (Azuonwu, Erhabor, & Frank-Peterside, 2011; Frank et al., 2013; Ramjee & Gouws, 2002). While few studies have looked at HIV testing among truck drivers, the South Africa study found that only 38.2% had ever tested for HIV (Delany-Moretlwe et al., 2014), and a 2009 study in a clinic at a truck stop in Mozambique found that only 25% of participants accepted HIV testing when offered and, and of those, 27% tested HIV+ (Lafort et al., 2010).

Truck drivers in Africa are considered a key population due to their high HIV risk and unmet need for services (International Labor Organization, 2005; South African National AIDS Council [SANAC], 2011; “Tackling HIV on Kenya’s transport corridors”, 2013). Health clinics targeting truck drivers now appear along many major transport routes (“Ethiopia Operational Plan report FY 2013, 2014; Lafort et al., 2010; North Star Alliance, 2016; “The trucking wellness program”, 2008), but in 2015, the North Star Alliance reported that in only 18% of the 253,227 client-visits at their 36 roadside wellness clinics across Africa was HIV testing accepted (North Star Alliance, 2016), while Trucking Wellness reported that only 19.5% of the 44,582 client visits in their 22 clinics in South Africa included HIV testing (Trucking Wellness, 2016). This suggests that even when healthcare services are targeted to truck drivers in a convenient form, HIV test uptake may remain suboptimal.

In 2012, the United States Food and Drug Administration (FDA) approved a rapid self-administered oral HIV test kit for at-home use (McNeil, 2012). Although the test is currently unavailable in most African countries, numerous studies have found self-administered HIV testing to be acceptable in African populations (Choko et al., 2011; Kalibala et al., 2014; Kelvin et al., 2016; Kurth et al., 2014, 2016; Ochako, Vu, & Peterson, 2014; Pant Pai et al., 2013; “A short technical update on self-testing for HIV”, 2014). Given the need to improve HIV testing rates to achieve the UNAIDS 90-90-90 goal, the first of which aims for 90% of those HIV-infected knowing their status (UNAIDS, 2014), it is imperative to assess new HIV testing modalities in key populations. Therefore, we conducted a randomized controlled trial comparing HIV test uptake by truck drivers at two roadside wellness clinics in Kenya among those offered a choice of HIV testing methods, including self-administered oral testing, versus the offer of only the one standard testing option. This study was extremely timely as Kenya announced the roll-out of self-testing kits in May 2017, about one year after this study was completed (UNAIDS, 2017).

## Materials and methods

### Setting

Participants were recruited from two North Star Alliance (NSA) roadside wellness clinics in Kenya. The NSA runs a total of 36 clinics in Africa, eight of which are in Kenya, which are open in the evenings to accommodate truck drivers’ work schedules. These clinics offer primary and secondary healthcare, including screening and treatment for sexual transmitted infection and HIV as well as for chronic diseases such as hypertension (North Star Alliance, 2016; Regondi et al., 2013). The two study clinics were selected because of their location in Nakuru county, which has among the highest HIV prevalence in the country (Kenya Ministry of Health, 2014; National STI and AIDS Control Programme [NASCOP], 2014). Together the clinics serve about 400 clients weekly, 30% of whom are truck drivers. Clients are offered HIV testing at every clinic visit and about 60% of truck driver clients accept testing, of whom about 1.5% test HIV+.

### Recruitment

All male truck drivers who visited the clinics during the recruitment period (October through December 2015) were informed of the research study by the receptionist. If interested, they were referred to a fieldworker for information and eligibility screening. The eligibility criteria were: (1) ≥ 18 years old, (2) male, (3) work as a truck driver, (4) reside in Kenya, (5) speak English or Kiswahili, (6) self-reported HIV-negative or unknown HIV status, (7) able to sign the consent form, and (8) willing to receive payment of participation fees via MPesa (a cell-phone-based money transfer system). Participants were told that study participation involved the completion of two questionnaires on the day of recruitment (baseline visit) and 6 months later, a phone-based questionnaire, which included questions about themselves, their lifestyle, including sexual behavior, and their thoughts about and experiences with HIV testing. The study was described to participants as being about HIV testing experiences and preferences and that HIV testing would be offered, as at any NSA clinic visit, but their decision about testing would not impact healthcare services or study eligibility. Participants were not informed about the specific research question or the fact that they would be randomized to different HIV testing options in order to avoid bias. In this paper we present the results regarding HIV testing at the baseline visit. The study procedures were approved by the City University of New York Institutional Review Board, the Kenya Medical Research Institute Ethics Committee, and the University of KwaZulu-Natal Biomedical Research Ethics Committee.

### Randomization and interviews

Participants completed a baseline interview to collect information about demographic background, HIV testing history and sexual risk behavior. Upon completing the baseline interview, the fieldworker opened a sealed envelope with the randomization assignment. Participants were randomized on a 1:1 basis to either the SOC arm or the Choice arm, stratified on clinic. Based on the randomization assignment, participants were offered HIV testing and, if accepted, underwent pre- and post-test counseling procedures. Following HIV testing (or test refusal), a second short interview was conducted asking about reasons for the decision to test or not and, for those in the Choice arm, why they selected the test they did. Data were collected on paper and taken to the NSA Nairobi office and entered into a REDCap database (Harris et al., 2009). Participants received 270 Kenyan Shillings (KES) (about $3 US) for completing the baseline interview and another 270 KES upon completion of the second interview following HIV testing or refusal to compensate them for their time in completing the interview.

### Study arms

#### Standard of care arm

Participants randomized to the SOC arm were offered the provider-administered blood-based (finger prick) rapid HIV test used in all NSA clinics (Colloidal Gold test) (World Health Organization, 2013).

#### Choice arm

Participants randomized to the Choice arm were offered the choice between (1) the SOC test or (2) supervised self-administered oral rapid HIV testing, and those who refused both options were then offered (3) a self-administered oral rapid HIV test kit to take for use outside of the clinic (home use).

The self-administered oral rapid HIV test kit used for this study was the FDA-approved OraQuick© In-Home HIV Test manufactured by OraSure (McNeil, 2012), but packaged for use in Kenya with pictorial and written instructions in English and Kiswahili. The Kenyan version of the test kit is smaller than the kit sold in the United States to reduce waste, and can fit in a pocket for confidentiality.

All clients in the Choice arm were given a demonstration of the self-test kit and explanation of the procedures before they made their decision. Participants who chose supervised self-testing in the clinic administered the test with the provider present. They were told to follow the instructions provided with the test kit, but that they could ask the provider questions while testing, and if they were doing something incorrectly, the provider would let them know the correct procedure. When the test results were ready, the participant could chose to view the test results alone or with the provider present to help with interpretation. If the participant wanted to view the results alone, the provider would leave the room and return for posttest counseling only after the participant had discarded the used test kit in its packaging to maintain privacy. During posttest counseling, the participant was encouraged, but not forced, to disclose the test results. Those who did not disclose the test result were to be given posttest counseling information for both possible scenarios (positive or negative HIV test) and referrals in case they were needed.

Clients in the Choice arm who refused both HIV testing options in the clinic were then offered a self-test kit for home use. Clients who chose to take a test kit received pretest counseling in the clinic and were instructed to use the test within three days and send a text message to the HIV counselor right after they completed the test to receive a call-back for posttest counseling, any needed referrals and to complete the second interview. Clients who took a test kit but did not text after three days were contacted by study staff and, if they reported having used the test, posttest counseling was provided. As with self-testing in the clinic, participants were encouraged to disclose the test result during posttest counseling and the content of the counseling depended on whether or not the results were disclosed.

### Sample size and power

We estimated that if the testing rate in the SOC group was 60%, as expected based on the testing rates in the clinics before the study, we would have 80% power to detect an odds ratio of 1.7 at a 2-sided alpha of 0.05 with a sample of 150 in each arm. The primary outcome was HIV testing in the clinic and the secondary outcome was HIV testing at baseline, including taking a test kit for home use.

### Statistical analysis

We described the sample overall and compared characteristics by randomization arm. To assess the statistical significance of differences by randomization arms, we used Pearson’s chi-square tests (or Fisher’s exact tests if any cell counts were < 5) for categorical variables and Mann-Whitney U tests for numeric variables. We then calculated Mantel Haenszel odds ratios for HIV test uptake by study arm adjusted for clinic (strata used in the randomization scheme). Finally, we reported frequencies for which HIV test was selected by those in the Choice arm and the reasons given for the selection, as well as the HIV test results for all participants. All statistical tests were two-sided at alpha = 0.05 and conducted using SPSS version 22 (Chicago, IL).

## Results

### Description of the sample

**Figure 1.**
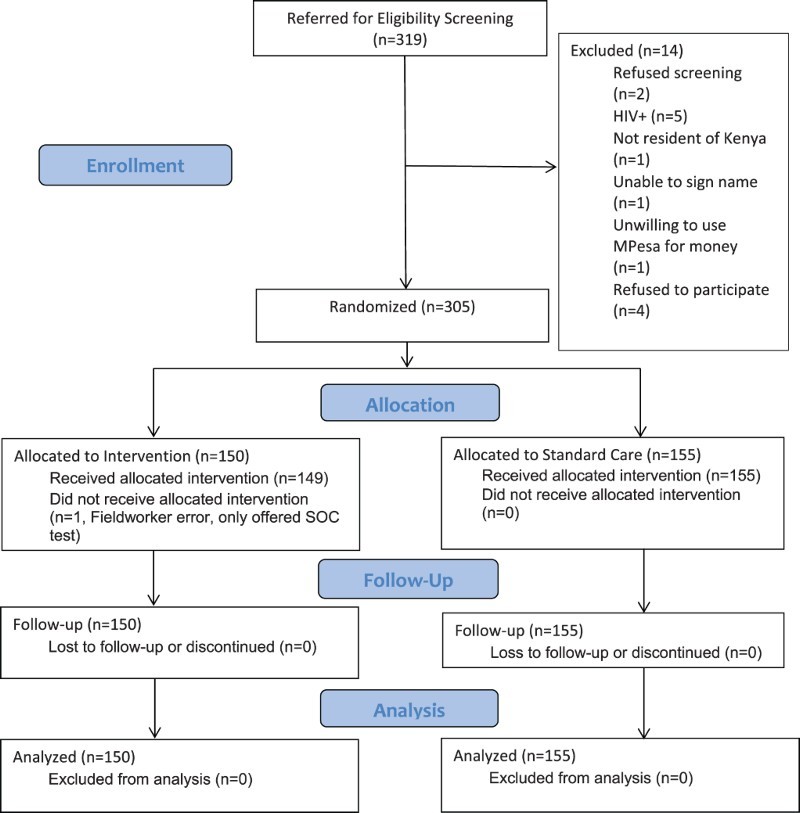
Flow of study participants (Consort Flowchart).

We screened 319 potential participants, of whom 305 were eligible and willing to participate (Figure 1). All participants were male and of black African race (data not shown). Their average age was 37.0 years. Nearly two-thirds (64.3%) had not completed secondary school and 27.8% earned less than 24,000 KES per month (about $235 US). Participants had worked as truck drivers for an average of 8.7 years. On average, the participants had spent 21.6 of the past 30 nights away from home due to work (Table 1).

**Table 1.** Descriptive statistics for the sample overall and by randomization arm.

Variable	Total, *n* (Column %)	SOC Arm, *n* (Row %)	Choice Arm, *n* (Row %)	*P*-value chi-square test, unless otherwise specified
Total	305	155 (50.8%)	150 (49.2%)	NA
Clinic where recruited				0.787
Clinic 1	144 (47.2%)	72 (46.5%)	72 (48.0%)	
Clinic 2	161 (52.8%)	83 (53.5%)	78 (52.0%)	
Age in years				0.989^a^
Mean (SD)	37.0 (7.9)	36.9 (8.0)	37.2 (7.8)	
Median (range)	36.0 (21.0–62.0)	35.0 (21.0–60.0)	37.0 (24.0–62.0)	
High school graduate				0.417
No	196 (64.3%)	103 (66.5%)	93 (62.0%)	
Yes	109 (35.7%)	52 (33.5%)	57 (38.0%)	
Mean trucking income per month (Kenyan Shillings)				0.074
8,000–15,999 KES	15 (5.2%)	12 (8.1%)	3 (2.1%)	
16,000–23,999 KES	65 (22.6%)	33 (22.3%)	32 (22.9%)	
24,000–55,000 KES	208 (72.2%)	103 (69.6%)	105 (75.0%)	
Number of years worked as truck driver				0.650^a^
Mean (SD)	8.7 (7.1)	9.0 (7.8)	8.4 (6.3)	
Median (range)	6.7 (1.0–38.9)	6.7 (1.0–38.9)	6.7 (1.0–37.0)	
Clinic is on usual trucking route				0.573
No	51 (16.8%)	24 (15.6%)	27 (18.0%)	
Yes	253 (83.2%)	130 (84.4%)	123 (82.0%)	
Number of nights away from home in the past 30 days				0.495^a^
Mean (SD)	21.6 (5.6)	21.3 (5.9)	21.8 (5.3)	
Median (range)	22.5 (0.0–30.0)	22.0 (0.0–30.0)	23 (2.0–30.0)	
Came to clinic specifically for HIV testing				0.365
No	173 (56.7%)	84 (54.2%)	89 (59.3%)	
Yes	132 (43.3%)	71 (45.8%)	61 (40.7%)	
Sexually active in the past 6 months				0.116^b^
No	6 (2.0%)	1 (0.7%)	5 (3.4%)	
Yes	295 (98.0%)	152 (99.3%)	143 (96.6%)	
Married (legal or common law)				0.999
No	51 (16.9%)	26 (16.9%)	25 (16.9%)	
Yes	251 (83.1%)	128 (83.1%)	123 (83.1%)	
Has other regular partner(s) on the trucking route				0.619
No	163 (53.4%)	85 (54.8%)	78 (52.0%)	
Yes	142 (46.6%)	70 (45.2%)	72 (48.0%)	
Paid for sex in the past 6 months				0.789
No	126 (44.1%)	65 (43.3%)	61 (44.9%)	
Yes	160 (55.9%)	85 (56.7%)	75 (55.1%)	
Always used condoms when had sex in the past 6 months (among those who had sex)				0.358
No	250 (85.9%)	127 (84.1%)	123 (87.9%)	
Yes	41 (14.1%)	24 (15.9%)	17 (12.1%)	
Ever tested for HIV before				0.259
No	25 (8.2%)	10 (6.5%)	15 (10.0%)	
Yes	280 (91.8%)	145 (93.5%)	135 (90.0%)	
Number of years since last HIV test among those ever tested				0.934^a^
Mean (SD)	1.1 (1.6)	1.0 (1.4)	1.1 (1.9)	
Median (range)	0.5 (0.1–12.0)	0.5 (0.1–7.4)	0.5 (0.1–12.0)	
Ever self-tested for HIV among those who ever tested				0.499^b^
No	276 (99.3%)	142 (98.6%)	134 (100.0%)	
Yes	2 (0.7%)	2 (1.4%)	0 (0.0%)	

^a^Mann-Whitney U test.

^b^Fisher’s exact test.

Almost all participants (98.0%) reported being sexually active in the past 6 months. Most (83.1%) were married, 46.6% reported that they had one or more regular partners along their route in addition to a wife or girlfriend at home, and 55.9% also reported having paid for sex in the past 6 months. Only 14.1% of participants reported always using condoms during sex in the past 6 months. Nearly all participants (91.8%) reported that they had previously tested for HIV, and the mean time since last HIV test was 1.1 years. There were no significant differences by randomization arm (Table 1).

### Impact of the intervention

Overall, 76.4% accepted HIV testing in the clinic. In the intent-to-treat analysis, those in the Choice arm had 1.5 times higher odds of accepting HIV testing in the clinic compared to those in the SOC arm. However, this difference was only of borderline significance (*p* = 0.189). One participant in the Choice arm was erroneously only offered the SOC test. When the data were analyzed per protocol by moving this person into the SOC arm, these results did not change (OR = 1.5, *p* = 0.196) (Table 2).

**Table 2.** HIV test uptake overall and by intervention status under intent-to-treat (i.e., by randomization assignment) and per protocol (i.e., by what was actually offered).

	Total, *n* (%)	SOC arm, *n* (%)	Choice arm, *n* (%)	Mantel Haenszel OR (95% CI) adjusting for strata	Mantel Haenszel *p*-value
Total as randomized	305 (100%)	155 (50.8%)	150 (49.2%)	NA	NA
Total per protocol	305 (100%)	156 (51.1%)	149 (48.9%)	NA	NA
Tested in clinic (intent-to-treat analysis)					
Yes	233 (76.4%)	113 (72.9%)	120 (80.0%)	1.5 (0.9–2.7)	0.189
No	72 (23.5%)	42 (27.1%)	30 (20.0%)	NA	NA
Tested in clinic (per protocol analysis)^a^					
Yes	233 (76.4%)	114 (73.1%)	119 (79.9%)	1.5 (0.9–2.7)	0.196
No	72 (23.6%)	42 (26.9%)	30 (20.1%)	NA	NA
Tested either in clinic or took test kit for home use (intent-to-treat analysis)					
Yes	244 (80.0%)	113 (72.9%)	131 (87.3%)	2.8 (1.5–5.4)	0.002
No	61 (20.0%)	42 (27.1%)	19 (12.7%)		
Tested either in clinic or took test kit for home use (per protocol analysis)^a^					
Yes	244 (80.0%)	114 (73.1%)	130 (87.2%)	2.8 (1.5–5.4)	0.002
No	61 (20.0%)	42 (26.9%)	19 (12.8%)	NA	NA

^a^One participant in the choice arm was only offered the SOC HIV test.

An additional 11 participants in the Choice arm accepted HIV testing when offered a test kit for home use after refusing testing in the clinic, all of whom reported they used the test during the follow-up phone-interview, bringing the total tested to 80.0%. When including self-testing at home, those in the Choice arm had 2.8 times the odds of HIV testing compared to those in the SOC arm and the difference was statistically significant (*p* = 0.002). In the per protocol analysis, these results did not change (OR = 2.8, *p* = 0.002) (Table 2).

Twenty-five participants reported never having been tested. Overall, 11/15 (73.3%) of those in the Choice arm tested compared to only 5/10 (50.0%) in the SOC arm tested, with an OR = 4.2 (*p* = 0.280) after adjusting for clinic. Four (35.3%) of those who tested in the Choice arm chose the SOC test, six (54.5%) chose supervised self-testing in the clinic, and one (0.9%) took a test kit to use outside of the clinic (Data not shown).

### HIV test selected by those in the choice arm

Of the 130 participants offered a choice in testing methods and who tested, 35 (26.9%) chose the SOC test, 84 (64.6%) chose supervised self-testing in the clinic, and another 11 (8.5%) took a test kit for home use. Among those who chose the SOC test, the most common reasons mentioned were that they prefer a provider administer or interpret the test for them (80.0%), they prefer a blood test over an oral test (60.0%), that they were not confident that they could administer the test correctly (17.1%), or they trust that the provider would administer the test correctly (14.3%). Among those who chose supervised self-testing in the clinic, the most common reasons were that they were curious to try the new test (89.3%), they felt confident that they could administer the test correctly themselves (25.0%), they prefer to administer the test themselves (15.5%) and that they prefer an oral test (15.5%). Among those who took a test kit for use outside of the clinic, the most common reasons mentioned were that they prefer to administer the test themselves (90.0%), felt confident that they could administer the test correctly themselves (45.5%), prefer to be with their partner, family or friends when testing (45.5%), or prefer to be alone when testing (36.4%), prefer an oral test (27.3%), and that they did not have time to test in the clinic (27.3%) (Table 3).

**Table 3.** HIV test used and reasons given for the test choice among those offered choices who agreed to test (*n* = 130).

Reason for test selected	SOC test, *n* (%)	Supervised self-administered rapid oral test in the clinic, *n* (%)	Self-administered rapid oral HIV test take for home use, *n* (%)^a^
Total	35 (26.9%)	84 (64.6%)	11 (8.5%)
Prefer provider to administer/interpret the test	28 (80.0%)	NA	NA
Prefer to administer/interpret the test myself	NA	13 (15.5%)	10 (90.9%)
Trust the provider can administer the test correctly	5 (14.3%)	NA	NA
Do not trust the provider to administer/interpret the test correctly	NA	1 (1.2%)	0 (0.0%)
Not confident that I could do the test correctly myself	6 (17.1%)	NA	NA
Feel confident that I can administer the test myself correctly	NA	21 (25.0%)	5 (45.5%)
Trust the provider to keep the results confidential	4 (11.4%)	NA	NA
Do not trust the provider to keep the results confidential	NA	1 (1.2%)	0 (0.0%)
Prefer to be the only one who knows my results	NA	1 (1.2%)	1 (9.1%)
Prefer to have someone with me when testing	1 (2.9%)	NA	NA
Prefer to be alone when testing	NA	1 (1.2%)	4 (36.4%)
Prefer to be with a partner or loved one when testing	NA	NA	5 (45.5%)
Feel uncomfortable in clinic settings	NA	NA	1 (9.1%)
Prefer a blood test	21 (60.0%)	NA	NA
Prefer an oral test	NA	13 (15.5%)	3 (27.3%)
Wanted to try the new test/curious about the new test	NA	75 (89.3%)	0 (0%)
Did not have time to stay at the clinic to test	NA	NA	3 (27.3%)

^a^Only offered to those in the choice arm who refused both in-clinic HIV testing options.

### HIV test results

Two participants tested positive for HIV and both were in the SOC arm (1.8%). All of the participants in the Choice arm who self-tested disclosed their test results to the counselor and all those test results were negative based on observation of the counselor for those who self-tested in the clinic and viewed their results with the counselor (*n* = 82) or based on the report of the participant for those who tested outside of the clinic (*n* = 11) or in the clinic but viewed their results alone (*n* = 2). This gives an HIV prevalence of 0.7% for the study participants (Table 4).

**Table 4.** HIV test results for those who tested by randomization arm.

	SOC Arm, *n* (%)	Choice Arm, *n* (%)
Total who tested	113 (100%)	131 (100%)
HIV-positive	2 (1.8%)	0 (0%)
HIV-negative	111 (98.2%)	131 (100%)
Refused to disclose (self-testers only)	NA	0 (0.0%)

## Discussion

To our knowledge, this is the first study to look at offering self-administered oral HIV testing to truck drivers, and one of the first to compare the offer of HIV testing choices versus a single option in any population. We found that truck drivers offered a choice of HIV testing methods were more likely to test compared to those offered only the SOC test. This difference did not reach statistical significance when only looking at testing in the clinic (OR = 1.5, *p* = 0.189), but it was significant when including taking a test kit for use at home (OR = 2.8, *p* = 0.002). Importantly, the additional 11 people who tested at home had already refused both in-clinic HIV testing options and would not have tested at all if they had not been offered a test kit to take with them. The higher uptake of self-administered testing in the clinic (64.6%) and for use outside of the clinic (8.5%) compared to the SOC test (chosen by 26.9%) among those offered a choice suggests that truck drivers in Kenya are ready for self-testing, as has also been indicated by studies with other groups in Kenya (Heard & Brown, 2016).

Study participants in the Choice arm varied in which test they picked, and a fair proportion (26.9%) picked the SOC test. This suggests that self-administered oral HIV testing should not replace the current HIV testing options, which work for many and are preferred by some. Instead, by offering choices, people can select the HIV testing method that meets their needs and preferences. When asked why they chose the test they did, reasons were guided by individual preferences, for example, between a blood versus an oral test or confidence in being able to self-test. Among those who refused both in-clinic options, wanting to test with partners, family or friends and not having time to test in the clinic were two common reasons for accepting a test kit for home use later. A fair number of self-testers said they chose the self-test because they were curious about it, among other reasons, suggesting that some might go back to the SOC test in the future. However, by offering three different test options, participants were able to choose the test that best fits with their individual needs and social circumstances.

This study had a number of limitations. First, the HIV testing rate in the SOC arm (72.9%) was higher than the 60.0% expected and the 55.0% for all clients at these two clinics during the study period; thus the study was underpowered for some comparisons. The higher testing rate we observed may be related to our offering HIV testing immediately following a detailed baseline interview about HIV testing and sexual risk behavior, which may have motivated some to test who would not have tested otherwise. However this may also indicate that our sample was not representative of all NSA clients, as is suggested by an HIV+ rate (0.7%) among study participants that was lower than that usually found in these clinics (1.5%). The low HIV+ rate could also be an indication that some who self-tested at home (*n* = 11) or in the clinic but viewed their test results in private (*n* = 2), disclosing the result only during posttest counseling, tested HIV+ but told the provider that they had tested HIV−. There is no way to know whether these participants disclosed their true test result, although they were informed that they could chose not to disclose the result at all, which we would hope would be preferable to misreporting the result. In addition, there may have been some error or social desirability bias in self-reported measures, particularly around HIV testing history and past sexual behavior. However, our outcome was based on observation in all cases except for the 11 people who self-tested outside of the clinic, thus minimizing error in the main analysis. Finally, our results may not be generalizable to all truck drivers in Kenya or in other countries.

Our findings suggest that offering self-administered oral HIV testing as a choice, together with the current testing options, may increase HIV testing rates among truck drivers in Kenya. Additional research is needed to confirm the findings in a larger sample and to ascertain the best ways to make self-testing available to truck drivers and other populations in order to maximize the impact of this new HIV testing method toward achieving the first 90 (that 90% of those HIV-infected know their status) in the 90-90-90 goal (UNAIDS, 2014).
